# Autologous hematopoietic stem cell transplantation promotes connective tissue remodeling in systemic sclerosis patients

**DOI:** 10.1186/s13075-022-02779-w

**Published:** 2022-04-29

**Authors:** Djúlio C. Zanin-Silva, Maynara Santana-Gonçalves, Marianna Y. Kawashima-Vasconcelos, João R. Lima-Júnior, Juliana B. E. Dias, Daniela A. Moraes, Dimas T. Covas, Kelen C. R. Malmegrim, Leandra Ramalho, Maria Carolina Oliveira

**Affiliations:** 1 Center for Cell-based Therapy, Regional Hemotherapy Center of the Ribeirão Preto Medical School, University of São Paulo, Ribeirão Preto, Brazil; 2 Basic and Applied Immunology Graduate Program, Ribeirão Preto Medical School, University of São Paulo, Ribeirão Preto, Brazil; 3 Oncology, Stem cell and Cell-Therapy Graduate Program, Ribeirão Preto Medical School, University of São Paulo, Ribeirão Preto, Brazil; 4 Internal Medicine Graduate Program, Ribeirão Preto Medical School, University of São Paulo, Ribeirão Preto, Brazil; 5 Department of Immuno-Oncology, Beckman Research Institute City of Hope, Duarte, CA USA; 6 Department of Internal Medicine, Ribeirão Preto Medical School, University of São Paulo, Avenida dos Bandeirantes 3900, Ribeirão Preto, SP 14048-900 Brazil; 7 Department of Clinical, Toxicological and Bromatological Analysis, School of Pharmaceutical Sciences of Ribeirão Preto, University of São Paulo, Ribeirão Preto, Brazil; 8 Department of Pathology and Legal Medicine, Ribeirão Preto Medical School, University of São Paulo, São Paulo, Brazil

**Keywords:** Scleroderma and related disorders, Cell transplantation, Skin, Metalloproteinases, Extracellular matrix

## Abstract

**Background:**

Autologous hematopoietic stem cell transplantation (AHSCT) treats patients with severe and progressive systemic sclerosis (SSc). However, basic mechanisms associated with the therapeutic efficacy of the procedure are not entirely understood. We aimed to evaluate how AHSCT affects skin fibrosis in SSc patients.

**Methods:**

Clinical data, serum, and skin samples from 39 SSc patients who underwent AHSCT were retrospectively evaluated. Skin biopsies were analyzed by immunohistochemistry with anti-MMP-1, -MMP-2, -MMP-3, -MMP-9, -TIMP-1, -α-SMA, -TGF-β, and -NF-κB p65 antibodies, and stained with hematoxylin and eosin and picrosirius red to assess skin thickness and collagen density, respectively. Serum samples were evaluated by Multiplex Assay for COL1A1, COL4A1, FGF-1, MMP-1, MMP-3, MMP-12, MMP-13, PDGF-AA, PDGF-BB, S100A9, and TIMP-1 levels and compared to healthy controls.

**Results:**

After AHSCT, SSc patients showed clinical improvement in skin involvement, assessed by modified Rodnan’s skin score (mRSS). Histologically, collagen density and skin thickness decreased after AHSCT. Immunohistochemical analyses showed increased expression of MMP-2, MMP-3, MMP-9, and TIMP-1 after AHSCT, whereas expression of NF-κB p65 decreased. At baseline, serum levels of COL4A1 and S100A9 were higher than in healthy controls. Serum levels of S100A9 normalized after AHCST in SSc patients compared to controls. Serum levels of PDGF-AA, PDGF-BB, TIMP-1, and MMP-1 decreased, while COL1A1 increased after AHSCT in SSc patients. No changes were detected in MMP-3, MMP-12, MMP-13, and FGF-1 serum levels after AHSCT.

**Conclusions:**

Our results suggest that the therapeutic effects of AHSCT on skin fibrosis are related to changes in molecules associated with connective tissue maintenance and inflammation in SSc.

**Supplementary Information:**

The online version contains supplementary material available at 10.1186/s13075-022-02779-w.

## Background

Systemic sclerosis (SSc) is an autoimmune disease marked by immunological deregulation, vasculopathy, and fibrosis of the skin and internal organs [1]. Autologous hematopoietic stem cell transplantation (AHSCT) is an intensive immunosuppressive therapy that has been investigated as treatment for severe and progressive SSc patients. Stem cell transplantation leads to the eradication of autoreactive immune cells and enables the reconstitution of a more tolerant immune system that contributes to clinical improvement [2]. Clinical and histological parameters of cutaneous fibrosis improve in SSc patients after AHSCT, indicating that the procedure affects immune pathways associated with fibrosis [1, 3].

Fibroblasts are the primary cells implicated with fibrosis: a process that involves increased production of extracellular matrix components (ECMs), such as collagen, and modifies connective tissue structure [1]. In SSc, fibroblasts present an activated phenotype (myofibroblasts), characterized by expression of alpha-smooth muscle actin (α-SMA), resistance to apoptosis, and increased synthesis of collagen, transforming growth factor-beta (TGF-β), and different types of ECMs [4]. Remodeling of ECMs is also compromised in the disease, with deregulated expression of metalloproteinases (MMPs) and their inhibitors [5].

Here, we aimed to investigate if AHSCT modifies the expression of molecules associated with fibrosis and connective tissue homeostasis in the skin and serum of SSc patients and how these results correlate with clinically detectable cutaneous fibrosis.

## Patients and methods

### Patients and clinical follow-up

Patients with severe and progressive SSc, who underwent AHSCT at the Ribeirão Preto Medical School (University of São Paulo, Brazil), were retrospectively evaluated and considered for this study. All patients transplanted from 2011 to 2016 were included, except four children under 16 years of age and two patients that did not reach the 12-month follow-up after AHSCT (one transplant-related death and one patient discontinued follow-up). Thirty-nine of these patients had available skin and/or serum samples from both pre and post-transplantation time points. Patients fulfilled the 1980 American College of Rheumatology (ACR) and the 2013 ACR/European League against Rheumatism (EULAR) classification criteria for SSc.

Detailed eligibility criteria for AHSCT and the transplant protocol have been previously described [6]. Indications for AHSCT included diffuse skin involvement or interstitial lung disease, with worsening of mRSS or pulmonary function tests, respectively, despite conventional immunosuppressive treatment. As a brief description of the transplant procedure, the autologous hematopoietic progenitor cells were mobilized from the bone marrow to the peripheral blood using a combination of 2 g/m^2^ intravenous cyclophosphamide plus subcutaneous injections of granulocyte-colony stimulating factor (G-CSF). Progenitor cells were harvested by apheresis and immediately cryopreserved, unmanipulated. Then, patients received the conditioning regimen, consisting of 200 mg/kg intravenous cyclophosphamide plus rabbit anti-thymocyte globulin, divided into five consecutive days of infusions. Three of the 39 patients received an alternative regimen of 120 mg/m^2^ Fludarabine and 120 mg/m^2^ Melphalan plus rabbit anti-thymocyte globulin due to baseline cardiac involvement. This alternative regimen is intended to avoid possible cyclophosphamide-induced cardiotoxicity in susceptible patients. Subsequently, the autologous progenitor cells were thawed at the bedside and infused intravenously. Patients remained in isolated protection until leukocyte engraftment (approximately 9–10 days after cell infusion) and were discharged from the hospital. Patients were then followed at the outpatient clinic for clinical and immunological outcomes.

Patients were evaluated before (baseline) and at 12 months after AHSCT. Baseline assessments were performed shortly before the procedure and were completed at a median (range) interval of 6 (3–18) days before the hematopoietic progenitor cell mobilization regimen. Ongoing immunosuppressive treatment was stopped immediately before beginning the evaluations. Routine clinical assessments were retrieved from patient medical records. They included modified Rodnan’s skin score (mRSS), complete blood counts, immunological and biochemical tests, echocardiography, computed tomography of the lungs, and pulmonary function tests. Patients collected blood and skin samples stored for future analysis at each time point, as detailed in the following sections. A group of sex- and age-matched healthy volunteers (healthy controls) donated blood samples at a single time point for analysis of serum markers.

Patients eligible for AHSCT signed informed consent forms agreeing to undergo transplantation. Upon enrollment for transplantation, additional written informed consent for blood and skin biopsy collection was obtained from all patients. The local ethics board (Comitê de Ética em Pesquisa do Hospital das Clínicas da Faculdade de Medicina de Ribeirão Preto, Ribeirão Preto, Brazil) approved the study protocol (number 71204717.6.0000.5440) on December 13, 2017. Due to the study’s retrospective design, the review board waived patient-signed consent for publication, and patient details were de-identified. The reporting of this study complies with the STROBE statement [7].

### Skin biopsies

Before transplantation, patients underwent punch biopsies (3–4 mm) of skin from the dorsal mid-forearm under local anesthesia. The subsequent 12-month biopsies (after AHSCT) were collected from areas adjacent to the first on the same arm. Skin samples were fixed in 10% buffered formalin and subsequently included in paraffin. Sections approximately 5 μm thick were cut from formalin-fixed blocks. Coded slides were deparaffinized in xylol, hydrated in ethanol and water gradient concentration. For skin thickness and collagen density analyses, sections were stained with hematoxylin and eosin (H&E) and picrosirius red, respectively. Immunohistochemistry was performed using 4 μm-thick sections. After deparaffinization, ethanol gradient, and water, samples underwent heat-induced antigen retrieval and blocking of nonspecific binding with protein block (Spring Bioscience, USA). We used immunohistochemistry to evaluate the tissue expression of different molecules associated with connective tissue remodeling within the skin structure. Tissue sections were stained with antibodies against MMP-1, MMP-2, MMP-3, MMP-9, TIMP-1 (1:100, Santa Cruz Biotechnology, USA), α-SMA (1:100, Abnova, Taiwan), TGF-β (1:100, R&D Systems, USA), and NF-κB p65 (1:100, Santa Cruz Biotechnology, USA). Sections were washed with TBST (TRIS-buffered saline) and incubated with fluorophore-conjugated antibodies (REVEAL Complement, Spring Bioscience) followed by HRP (horseradish peroxidase) Conjugate (Spring Bioscience). The reaction was developed using NovaRED chromogen (Vector Labs, USA), and sections were counterstained by a 40-s incubation in hematoxylin. Lastly, slides were washed and mounted. All specimens were incubated with an isotype-matched control antibody under identical conditions for negative controls.

### Histological analysis

Sections were assessed using a Carl Zeiss LSM710 Observer Confocal System microscope (Carl Zeiss, Germany), and images were analyzed in ImageJ software (1.53 version; National Institutes of Health, USA), with a plug-in package developed by the McMaster University (Ontario, Canada). Analyses were performed on five randomly taken images by a single pathologist (LNR), blinded to patient identification and status. Skin thickness was measured in H&E preparations from the base of the dermis to the stratum corneum of the epidermis under × 20 magnification. The remaining (picrosirius red staining and immunohistochemistry) images were taken at × 5 magnification. Skin sections stained for collagen (picrosirius red) or immunohistochemistry were scanned and then blindly assessed for the percentage of the area marked with Sirius red labeling (red) or immunolabeling (red), respectively. Specifically, for α-SMA staining, the vessels and glands were excluded from the analysis through a subtraction system from the images [8]. In the analysis of the images, marked areas enable quantification of the expression of molecules bound to specific antibodies, as staining intensity is proportional to the concentration of the protein.

### Serum analyses

Whole blood samples were collected at baseline and 12 months after AHSCT and at a single point for healthy controls. Samples were spun at 1500 r.p.m. for 10 min at room temperature and then stored at − 80°C. Eleven molecules—MMP-1, MMP-3, MMP-12, MMP-13, TIMP-1, COL1A1 (collagen I alpha 1), COL4A1, PDGF-AA (platelet-derived growth factor), PDGF-BB, S100A9 (S100 calcium-binding protein A9), and FGF-1 (fibroblast growth factor-1)—related to fibrosis and connective tissue homeostasis were measured in the serum using Magnetic Luminex Assay (R&D System), according to manufacturer’s instructions. Briefly, in 96-well plates, standard solution, serum (diluted in a 1:2 or 1:50 proportion), and analyte-specific antibodies (pre-coated onto magnetic microparticles embedded with fluorophores) were added. Next, samples were incubated, washed, and a biotinylated detection antibody was added, followed by Streptavidin-PE solution. Lastly, the plate was read in a MAGPIX System instrument (Luminex Corporation, USA). Standard curves were created for each analyte, mean fluorescence intensities were determined, and final concentrations (pg/mL) were established.

### Statistical analysis

Clinical data were described as percentages, median (range), or mean (standard deviation). Data from clinical, histological, and serum assessments in SSc patients before and after AHSCT were tested for normality using the Shapiro-Wilk test and subsequently compared by paired t-test or Wilcoxon test. Serum analysis results from healthy controls and transplanted SSc patients and between groups of patients divided according to mRSS were compared by Student’s *t* test or Mann-Whitney test. Spearman’s test determined correlations. Data were analyzed, and figures were created using GraphPad Prism 8 (8.3.1 version; GraphPad Software Inc., USA). Significance was established at *p* < 0.05.

## Results

### Patient characteristics and clinical response to AHSCT

**Fig. 1:**
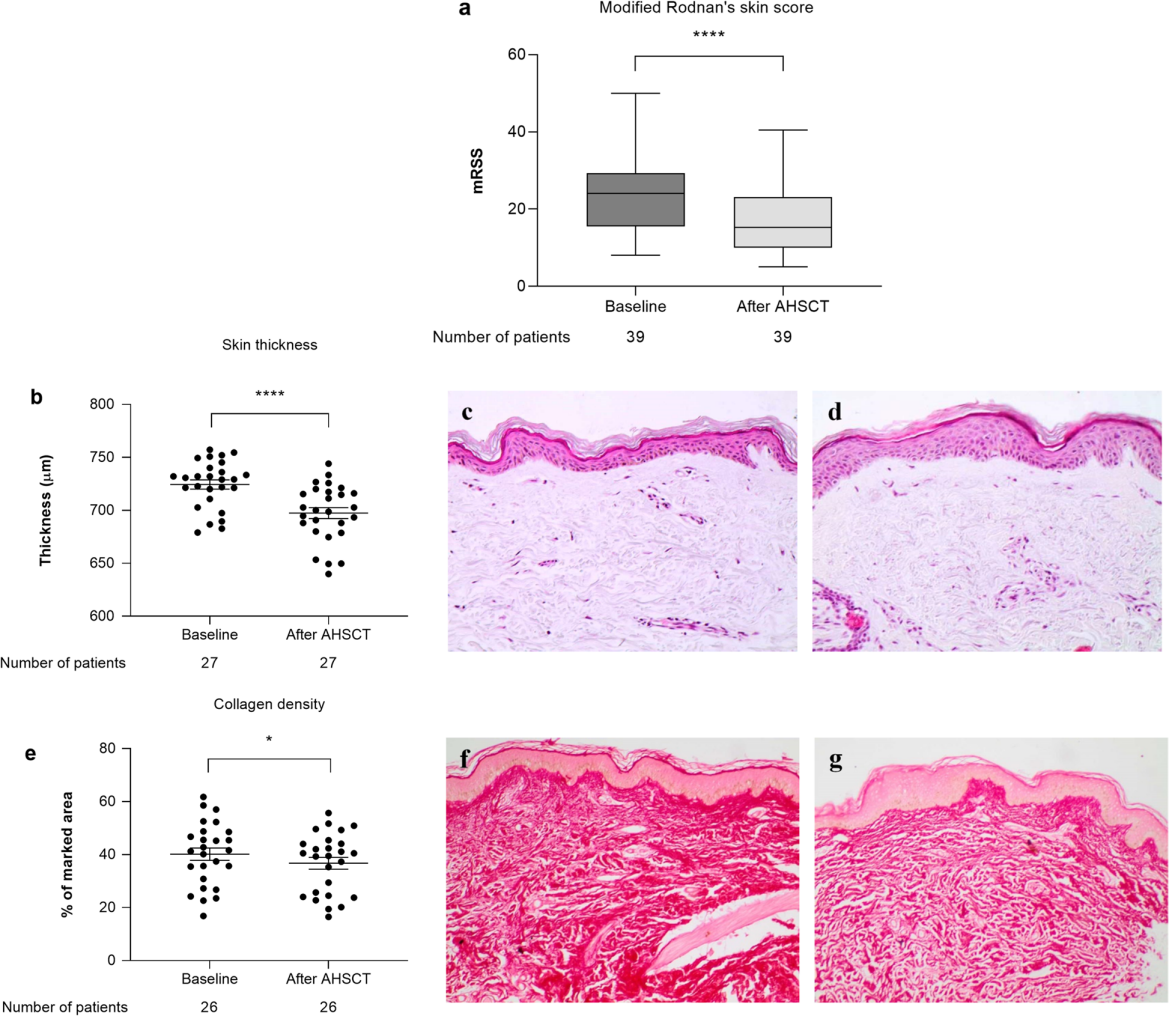
Clinical and histological aspects associated with skin fibrosis in SSc patients undergoing AHSCT. **a** Modified Rodnan’s skin score (mRSS) assessment. The boundaries of the boxes indicate the 25th and 75th percentiles; lines within the boxes indicate the median and the whiskers mark the 10th and the 90th percentiles. Plots show mean ± standard deviation (SD). ****p < 0.0001 comparing post-transplantation and baseline values. **b** Skin thickness measured in skin biopsies stained with H&E (μm) before and after AHSCT. Mean ± standard error of mean (SEM); ****p < 0.0001. **c**, **d** Photomicrographs from a representative systemic sclerosis patient, before (**c**) and after (**d**) AHSCT. **c** Thickening and hyalinization of connective tissue of the deep dermis, subcutaneous fat, and muscular fascia. Frequent atrophy of adnexal structures, thickening and luminal narrowing of small vessels, blunting of the dermis-subcutis interface, and diminished elastic tissue (H&E, × 20 magnification). **d** Reduction in collagen deposits in the dermis and higher frequency of cutaneous annexes in the dermis (H&E, × 20 magnification). **e** Collagen density (mean ± SME) in SSc skin biopsies stained with picrosirius red (% of the marked area), before and after AHSCT; *p < 0.05. **f**, **g** Photomicrographs from a representative systemic sclerosis patient, before (**f**) and after (**g**) AHSCT (red fibers, Sirius red staining, × 5 magnification). **f** Increased deposition of collagen in the dermis. **g** Reduction of collagen deposits in the dermis

Pre-transplantation characteristics from the 39 SSc patients with available samples are described in Table 1. All patients included in the study had the diffuse cutaneous subtype of SSc (dcSSc). At transplantation, the median (range) age and duration of non-Raynaud’s manifestations were 35 (18–59) and 2 (0.5–14) years, respectively, and 82% of the patients were female. Skin fibrosis, assessed by mRSS, improved from mean (SD) 24 (9) at baseline to 16 (8) at 12 months after AHSCT (Fig. 1a).

**Table 1:** Patient baseline characteristics and transplant details

Total number of adult patients	39
Median (range) age (years)	35 (18–59)
Gender (%)	32 (82%) female
Disease subtype	39 (100%) diffuse
Median (range) disease duration (years)^a^	2 (0.5-14)
Organ involvement:	
Skin, *n* (%)	39/39 (100%)
mRSS units, median (range)	24 (8–50)
Interstitial lung disease, *n* (%)	28/39 (71.79%)
Pulmonary hypertension, *n* (%)	3/39 (7.6 %) mild
Forced vital capacity, % of predicted, mean (SD)	74.68 (32.5)
Heart involvement, *n* (%)^b^	16/39 (40.9%)
Esophageal dysmotility, *n* (%)	36/39 (92.30%)
Kidney, *n*	0
Positive anti-Scl-70 antibodies, *n* (%)	26/39 (66.6%)
Previous immunosuppressive treatment:	
Methotrexate, *n* (%)	17/39 (43.1 %)
Cyclophosphamide, *n* (%)	29/39 (74.4 %)
Mycophenolate mofetil, *n* (%)	8/39 (20.5 %)
Rituximab, *n* (%)	2/39 (5.1 %)
Transplant regimen:	
Cyclophosphamide + ATG	36/39 (92.3%)
Fludarabine + Melphalan + ATG	3/39 (7.7 %)

Baseline (pre-transplant) clinical characteristics of patients enrolled in the study

*mRSS* modified Rodnan Skin Score, *IV* intravenous infusion, *ATG* anti-thymocyte globulin

^a^Disease duration from first non-Raynaud’s phenomenon clinical manifestation until transplant

^b^Patients with severe heart involvement were excluded from transplant

### AHSCT improves skin thickness, collagen density, and expression of markers associated with extracellular matrix remodeling and inflammatory pathways

**Fig. 2:**
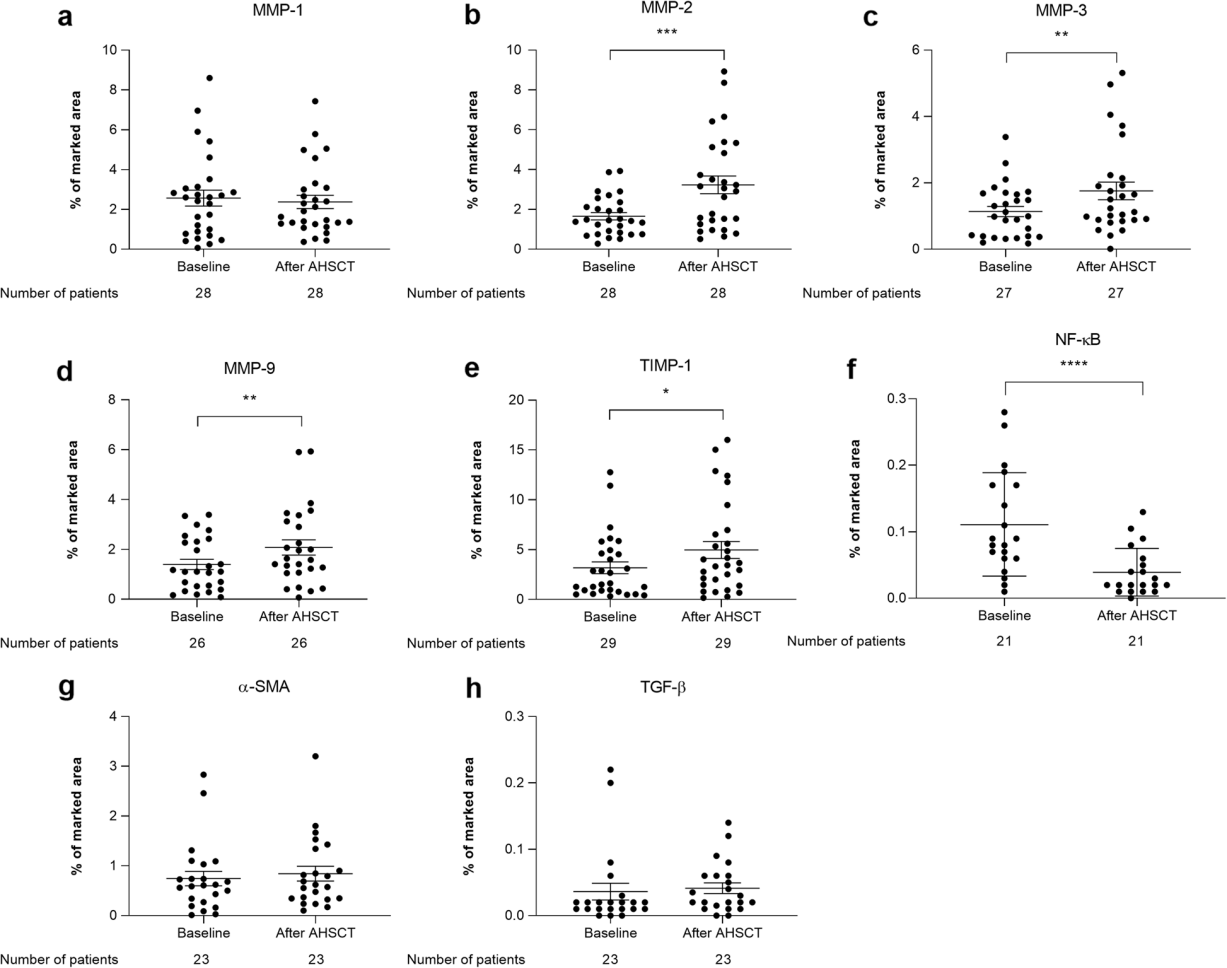
Expression of molecules associated with extracellular matrix remodeling and fibrosis in skin biopsies of SSc patients undergoing AHSCT. Mean ± standard deviation of skin sections marked by immunohistochemistry for **a** MMP-1, **b** MMP-2, **c** MMP-3, **d** MMP-9, **e** TIMP-1, **f** NF-κB p65, **g** α-SMA, and **h** TGF-β expression (% of marked area) in SSc skin biopsies. *p < 0.05. **p < 0.01. ***p < 0.001

When compared to the pre-transplantation (baseline) assessments, histological analysis of SSc skin biopsies showed a significant decrease in skin thickness (*p* < 0.0001) (Fig. 1b–d) and collagen density (*p* < 0.05) (Fig. 1e–g) after transplantation. Cutaneous expression of MMP-1 (*p* = 0.518) (Fig. 2a) did not change, but expressions of MMP-2 (*p* < 0.001) (Fig. 2b), MMP-3 (*p* < 0.01) (Fig. 2c), MMP-9 (*p* < 0.01) (Fig. 2d), and TIMP-1 (*p* < 0.05) (Fig. 2e) increased, while NF-κB expression decreased after AHSCT (Fig. 2f) (*p* < 0.0001). No changes in skin expression of the myofibroblast marker α-SMA (*p* = 0.822) (Fig. 2g) or the profibrotic cytokine TGF-β (*p* = 0.196) (Fig. 2h) were detected after AHSCT.

### AHSCT modifies serum concentrations of molecules linked to skin inflammation and fibrosis in SSc patients

**Fig. 3:**
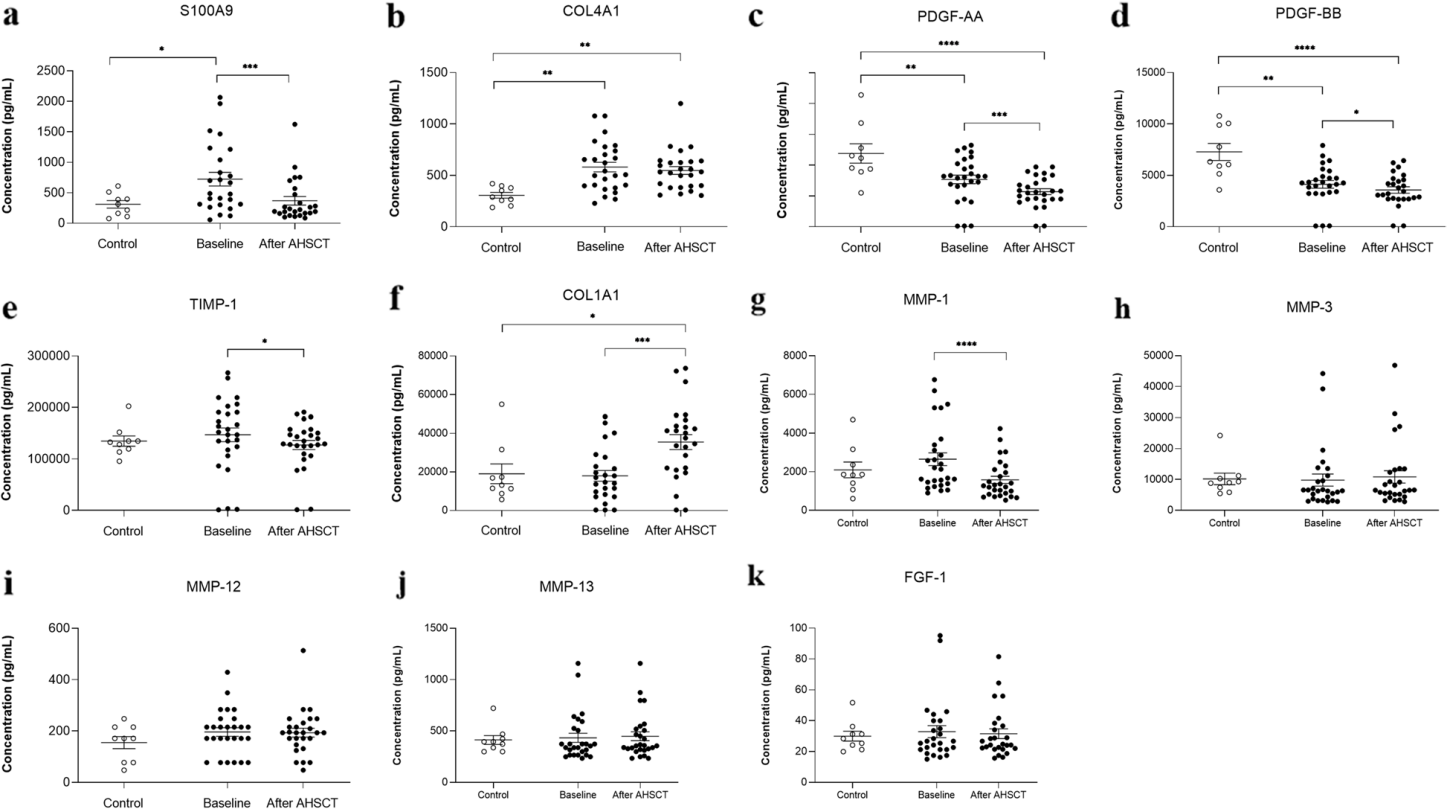
Serum levels of molecules associated with skin inflammation, fibrosis, and connective tissue maintenance in healthy controls and SSc patients at baseline and after AHSCT. Mean ± standard deviation of **a** S100A9, **b** COL4A1, **c** PDGF-AA, **d** PDGF-BB, **e** TIMP-1, **f** COL1A1, **g** MMP-1, **h** MMP-3, **i** MMP-12, **j** MMP-13, and **k** FGF-1 serum concentrations measured by Magnetic Luminex Assay. *p < 0.05. **p < 0.01. *** *p* < 0.001. ****p < 0.0001

Before transplantation, serum concentrations of S100A9 (*p* < 0.05) (Fig. 3a) and COL4A1 (*p* < 0.01) (Fig. 3b) were higher in SSc patients when compared to healthy controls. After transplantation, serum levels of COL4A1 in SSc patients did not change and remained higher than in healthy controls (*p* < 0.01) (Fig. 3b). Levels of PDGF-AA (Fig. 3c) and PDGF-BB (Fig. 3d) were significantly lower in SSc patients at baseline (*p* < 0.01) compared to controls, remaining lower after AHSCT (*p* < 0.0001). After transplantation, SSc patients presented a reduction in S100A9 (*p* < 0.001) (Fig. 3a) and TIMP-1 (*p* < 0.05) (Fig. 3e). Levels of S100A9 decreased and normalized after AHCST in SSc patients (Fig. 3a). PDGF-AA (*p* < 0.001) and PDGF-BB (*p* < 0.05) concentrations also decreased after AHSCT (Fig. 3a and b). COL1A1 levels increased (*p* < 0.001) in SSc patients after AHSCT compared to baseline and became higher than in healthy controls (*p* < 0.05) (Fig. 3f). MMP-1 (*p* < 0.0001) (Fig. 3g) concentrations decreased post-transplantation when compared to baseline. MMP-3 (*p* = 0.155) (Fig. 3h), MMP-12 (*p* = 0.638) (Fig. 3i), MMP-13 (*p* = 0.241) (Fig. 3j) and FGF-1 (*p* = 0.707) (Fig. 3k) serum levels did not change after transplantation in SSc patients and were not different than healthy controls.

### Transplant-induced changes in histological and serum markers correlate with clinically assessed fibrosis in SSc patients

At baseline, there were positive correlations between mRSS and serum levels of COL-IV (*r* = 0.63, *p* = 0.0004) and MMP-1 (*r* = 0.41, *p* = 0.03). Positive correlations were also found between mRSS and the percentage of marked areas, in the skin, for MMP-3 (*r* = 0.6, *p* = 0.01) and MMP-1 (*r* = 0.51, *p* = 0.04).

We also compared the variation (difference between values at 12 months and at baseline; Δ) between mRSS and each of the serological and histological markers, to determine if the clinical (mRSS) improvement of SSc patients after AHSCT correlated with changes in the analyzed parameters. In the histological analyses of the skin, there were positive correlations between ΔmRSS and changes in expression of TIMP-1 (ΔTIMP-1, *r* = 0.455; *p* < 0.01) and between ΔmRSS and changes in collagen density (ΔPicrosirius, *r* = 0.4822; *p* < 0.01) (Table S1). In the serum analyses, there were positive correlations between ΔmRSS and ΔS100A9 (*r* = 0.4913; *p* < 0.001), ΔPDGF-AA (*r* = 0.4701; *p* < 0.001), ΔPDGF-BB (*r* = 0.3115; *p* < 0.05), and ΔMMP-1 (*r* = 0.6051; *p* < 0.00001) concentrations and negative correlation between ΔmRSS and ΔCOL1A1 (*r* = -0.3529; *p* < 0.01) serum levels (Table S2).

To evaluate if the severity of skin involvement influenced the expression of fibrosis markers, we clustered the patients according to baseline skin involvement, using the mRSS of 20 points as the cut-off value to stratify groups [9]. Out of the 39 included patients, 15 were classified as mRSS ≤ 20 and 24 as mRSS > 20. Subsequently, groups were compared for baseline and post-transplantation levels of serum and skin markers (Tables S3 and S4, respectively). For serum markers, COL4A1 and PDGF-BB were higher in patients with mRSS > 20 than in those with mRSS ≤ 20 at baseline. After AHSCT, MMP-3, MMP-12, and FGF-1 were higher in patients with mRSS > 20 than in mRSS ≤ 20 (Table S3). MMP-13 serum levels were higher in the group of patients with mRSS ≤ 20 at baseline and remained higher at the post-AHSCT time point when compared to the mRSS > 20 group of patients. For skin markers, TGF-β was higher in patients with mRSS ≤ 20 than in those with mRSS > 20 at baseline and after AHSCT (Table S4). When differences (delta, Δ) between baseline and post-AHSCT from each group were compared for skin expression of immunohistochemistry markers, MMP-3 increased more in the group of patients with mRSS > 20 (mean percentage of marked area = 0.9787, SD = 0.114) than in those with mRSS ≤ 20 (mean = 0.1724, SD = 0.438; *p = 0.027*) (Table S4). For serum markers, only PDGF-BB levels decreased more in the group of patients with mRSS >20 (mean = − 2,011 pg/mL, SD = 4780) than in those with mRSS ≤ 20 (mean = -102 pg/mL, SD = 859; *p = 0.048*) (Table S3).

## Discussion

The main steps of AHSCT for SSc include ablation of autoreactive cells, followed by reconstitution of a renovated and tolerant immune system. Stem cell transplantation can modify the inflammatory components of the disease [10], and clinical response to AHSCT is associated with reduced autoreactivity, increased regulation, and modulation of the inflammatory environment [11]. Here, we demonstrated that AHSCT was also influential on mechanisms related to fibrosis, a key element in the pathogenesis of SSc.

Skin fibrosis is a hallmark of the disease and affects most SSc patients. High mRSS values indicate poor prognosis [12], and improved skin thickening over time is associated with increased survival [13]. In our patients, skin fibrosis, clinically assessed by mRSS, considerably decreased after AHSCT, agreeing with other reports of maximum skin improvement in the first year after transplantation [14, 15]. Our histological analyses showed that skin thickness and collagen density decreased in the 12 months that followed transplantation, corroborating reports of improved dermal fibrosis after AHSCT [3, 14].

Fibrosis is a process characterized by exacerbated synthesis and deposition of ECMs, especially collagen. In SSc, collagen staining on skin biopsies correlates with mRSS severity [8]. Indeed, we found a positive correlation between changes in collagen density and mRSS after transplantation, which may indicate that AHSCT effectively improves clinically assessed skin thickening and collagen turnover, and connective tissue remodeling.

Extracellular matrix remodeling releases specific tissue molecules into the peripheral circulation [16]. In the skin, about 85% of collagen fibers are type 1 [17]. Serum concentrations of COL1A1 increased after transplantation in our SSc patients. They remained high compared to healthy controls, suggesting sustained degradation and release of collagen type 1 into the peripheral blood during the one-year follow-up after AHSCT. Serum levels of COL4A1, however, remained higher than control levels before and after transplantation. Collagen type IV is present in basal membranes of blood vessels [18]. This type of collagen is found at increased serum levels in SSc patients, especially those with higher mRSS or with digital ulcers, an indicator of tissue and vascular damage [19]. Therefore, the sustainably high concentrations of COL4A1 in our patients may indicate the persistence of the SSc-associated vasculopathy after AHSCT, despite improvement of fibrosis, as previously reported [14].

In addition to collagen, AHSCT may also affect the metabolism of different ECMs. We demonstrated an increase in cutaneous expression of MMP-2, MMP-3, and MMP-9, which process a range of ECMs substrates, such as fibronectin, proteoglycans, and laminin [20]. Isolated dermal fibroblasts from SSc patients have decreased mRNA expression of MMP-1, MMP-2, and MMP-3 [20], supporting the hypothesis that AHSCT restores expression and catalytic activity of some MMPs, thereby increasing the degradation of ECMs in the skin. Indeed, in our study, MMP-3 expression in the skin increased post-AHSCT. In patients with higher mRSS, post-transplantation changes (delta) in MMP-3 expression on the skin were significantly higher than in patients with less skin involvement. However, in the serum, we did not detect changes in MMP-3 levels, which remained similar to healthy controls after AHSCT. These discrepant results between local and systemic evaluations (Supplementary Table S5) may indicate that AHSCT has distinct effects on separate disease compartments, as serum analyses may reflect fibrotic changes beyond the skin.

MMP-1 levels declined in the serum and did not change in patients’ skin after AHSCT. Although a previous study suggests tissue binding of MMP-1 as a possible explanation for the decrease in serum levels in SSc patients [21], we did not find evidence of increased expression on the skin. Further studies evaluating the enzymatic activity of MMP-1 may improve the understanding of these unexpected results.

On the other hand, TIMPs control the enzymatic activity of MMPs. Previous studies have shown that SSc patients have high concentrations of TIMP-1 in the serum, reflecting deficiencies in ECM degradation [21, 22]. Here, we observed a reduction of TIMP-1 serum concentrations after AHSCT, indicating that the blocking of MMP activity was reduced. After transplantation, expression of TIMP-1 increased in the skin biopsies of our patients, suggesting a local control of MMP functions. It is possible that TIMP-1 tissue levels increased in response to the elevation in MMPs expression and, perhaps, activity. Indeed, our correlation analyses showed that changes in mRSS were proportional to changes in skin expression of TIMP-1.

In different fibrotic diseases, increased ECMs synthesis occurs through myofibroblast activity [23], which can be regulated by TGF-β, a cytokine that promotes collagen synthesis and inhibits MMPs [24]. Michel et al. demonstrated a reduction in TGF-β serum levels 6 months after AHSCT in SSc patients [25]. Thereby, we believe that in our patients' skin, intracellular signalization of TGF-β may change after transplantation, similar to SMAD3. This intracellular protein exerts an essential function in transducing TGF-β signals, which are highly expressed in the skin of SSc patients [26].

Unexpectedly, the expression of α-SMA in our patients' skin did not change after AHSCT, which can be due to the already established fibrosis of SSc, when the tissue presents many collagen bands and has low cellularity [27]. In wound healing processes and systemic sclerosis, myofibroblasts express α-SMA temporarily during their differentiation, and staining decreases to undetectable levels as fibrosis progresses [28, 29]. Also, α-SMA may be expressed by other cells involved in the pathogenesis of SSc, such as endothelial cells [30], whose influence on disease activity is not completely controlled by AHSCT [31]. The expression of α-SMA and other markers and functional aspects of myofibroblasts are currently being evaluated by in vitro assays using isolated cells [32–34]. Similar strategies may be adopted in the future to investigate how transplantation affects these cells.

The substantial decrease of nuclear factor-kappa B (NF-κB) expression in our patients' skin indicates how AHSCT affects the inflammation-fibrosis axis. NF-κB is a transcription factor involved in multiple immunological processes and, when activated, induces the expression of cytokines, chemokines, and growth factors [35]. Activation of NF-κB is also associated with pro-fibrotic mechanisms, including the dysregulated proliferation of keratinocytes observed in SSc [36]. Keratinocytes in the epidermis secrete cytokines, growth factors, and chemokines and may be critical regulators of fibroblast function. Isolated scleroderma keratinocytes express high levels of NF-κB-regulated cytokines and chemokines [36]. In our patients, the decrease of NF-κB expression in the skin, coupled with normalization of S100A9 serum levels, may suggest that AHSCT affects epithelial cells involved with SSc pathogenesis, such as the keratinocytes.

S100A9 is an alarmin produced by activated keratinocytes, implicated in SSc pathogenesis [37, 38]. Under homeostatic conditions, S100A9 is stored in myeloid cells. However, it can be intensely upregulated during inflammatory processes in epithelial cells and osteoclasts [39]. High concentrations of S100A9 are present in the serum and in the bronchoalveolar lavage fluid of SSc patients, correlating with disease activity [40, 41]. At baseline, our patients had higher serum levels of S100A9 than healthy controls, but these levels decreased and normalized after AHSCT, suggesting an improvement in the inflammatory status. We also found a positive correlation between changes in mRSS values and S100A9 serum levels in our patients, reinforcing that AHSCT can modify inflammation-fibrosis signals. Recently, a phase I clinical trial evaluated the effects of Paquinimod, a molecule that inhibits S100A9 signalization, as a treatment for SSc patients [42]. Although no effects were observed on mRSS, chemokine (C-C motif) ligand 2 (CCL2) serum levels and mRNA levels in the skin decreased after administration of Paquinimod, indicating a favorable effect on mechanisms of inflammation and fibrosis associated with the disease.

Unlike the profibrotic molecules we evaluated, serum levels of PDGF-AA and PDGF-BB were significantly lower in SSc patients’ serum than in healthy controls, both at baseline and after AHSCT. Although PDGF is an important mitogen to fibroblasts [43], it also participates in angiogenesis, which is dysregulated in SSc [44]. Studies are controversial about PDGF levels in the peripheral blood of SSc patients, with results that range from not different to higher than those of healthy controls [25, 45, 46]. Moreover, pathogenic autoantibodies targeting PDGF receptors were found in the serum of SSc patients [47]. Further studies are warranted to understand a possible link between PDGF, fibrosis, and endothelial dysfunction in SSc.

Finally, we believe that the therapeutic effects of AHSCT result in changes in molecular mediators and different cell subsets involved with fibrosis in SSc. Nintedanib, a tyrosine kinase inhibitor indicated for the treatment of idiopathic pulmonary fibrosis and, more recently, interstitial lung disease secondary to SSc, has beneficial effects on the lungs but not on other disease manifestations such as skin fibrosis [48]. Similarly, pirfenidone, another drug used to treat idiopathic pulmonary fibrosis, failed to improve skin involvement in SSc [49]. These results suggest that each tissue has its unique microenvironment and pathogenic mechanisms dynamic, thus responding differently to treatments.

This study is limited by the retrospective design and short-term assessment of one year after AHSCT. Nevertheless, skin improvement is more evident within this 12-month timeframe, as shown by decreasing mRSS scores early after AHSCT [3, 14, 15, 26]. Another limitation is that the biopsies evaluate a small area of the skin and may not reflect the full presentation of the disease. However, all patients had the diffuse SSc subtype, which contributes to homogenizing of the study population, and we were able to include a large number of patients. Three of our patients had a disease duration of more than 10 years, indicating advanced fibrotic stages of the disease. Nevertheless, these patients presented mRSS, disease thickness, and biomarkers in the skin and serum that were not different from the remaining patients at baseline and after AHSCT, suggesting that connective tissue remodeling mechanisms induced by transplant operate even in patients with more advanced disease.

As a general approach to reverse tissue fibrosis, “sick” ECMs need to be removed or replaced by “healthy” ECMs [50]. Resolution of fibrosis is a dynamic process involving eliminating fibroproliferative stimuli associated with the immune system, removing transformed myofibroblasts, and degrading and clearance of ECMs [50]. Previous studies have shown that stem cell transplantation reverses fibrosis of the bone marrow in patients with myelofibrosis [51, 52], indicating that the procedure affects fibrosis-related mechanisms. We believe that our results may shed light on these mechanisms and stimulate further developments in therapeutic strategies for skin fibrosis in SSc, even beyond the transplantation scenario [53].

In conclusion, we demonstrated that AHSCT decreased skin fibrosis and modified the expression of molecules related to connective tissue maintenance and inflammation. We believe that, in addition to the primary mechanism for controlling immunological autoreactivity, AHSCT enables other therapeutic pathways that together contribute to the favorable clinical outcomes of SSc patients.

## Supplementary Information

**Additional file 1: Table S1**: Correlations between histological markers and modified Rodnan’s skin scores (mRSS).

**Additional file 2: Table S2**: Correlations between serum molecules and modified Rodnan’s skin score (mRSS).

**Additional file 3: Table S3**: Serum levels of molecules in SSc patients clustered according to severity of skin involvement.

**Additional file 4: Table S4**: Immunohistochemistry (skin) results in SSc patients clustered according to severity of skin involvement.

**Additional file 5: Table S5**: Summary of transplanted-induced changes in connective tissue and fibrosis-related molecules in the serum and skin of systemic sclerosis patients.

## Data Availability

The datasets generated and/or analyzed during the current study are not publicly available due to ethical/privacy reasons.

## Abbreviations

ACR: American College of Rheumatology
AHSCT: Autologous hematopoietic stem cell transplantation
ATG: Anti-thymocyte globulin
COL1A1: Collagen I alpha 1
dcSSc: Diffuse cutaneous form of systemic sclerosis
ECMs: Extracellular matrix components
EULAR: The European League Against Rheumatism
FGF-1: Fibroblast growth factor-1
G-CSF: Granulocyte colony-stimulating factor
H&E: Hematoxylin and eosin
IL: Interleukin
MMP: Metalloproteinase
mRSS: Modified Rodnan’s skin score
NF-κB: Nuclear factor-kappa B
PDGF: Platelet-derived growth factor
S100A9: S100 calcium-binding protein A9
SSc: Systemic sclerosis
TGF-β: Transforming growth factor-beta
TIMP: Tissue inhibitor of metalloproteinases
α-SMA: Alpha-smooth muscle actin
