# Supplementary material for: Autologous hematopoietic stem cell transplantation promotes connective tissue remodeling in systemic sclerosis patients
Source: Arthritis Res Ther. 2022 Apr 29;24:95. doi: 10.1186/s13075-022-02779-w (PMC9052524; doi:10.1186/s13075-022-02779-w)
Supplement: Supplementary file 1 — Additional file 1: Table S1: Correlations between histological markers and modified Rodnan’s skin scores (mRSS). [file 13075_2022_2779_MOESM1_ESM.docx]

| Correlation  (Spearman) | ΔmRSS  vs  ΔMMP-1 | ΔmRSS  vs  ΔMMP-2 | ΔmRSS  vs  ΔMMP-3 | ΔmRSS  vs  ΔMMP-9 | ΔmRSS  vs  ΔTIMP-1 | ΔmRSS  vs  Δα-SMA | ΔmRSS  vs  ΔH&E | ΔmRSS  vs  ΔPicrosirius | ΔmRSS  vs  ΔNF-κB | ΔmRSS  vs  ΔTGF-β |  |  |  |
| --- | --- | --- | --- | --- | --- | --- | --- | --- | --- | --- | --- | --- | --- |
| r | 0.1768 | 0.3320 | -0.1149 | 0.1365 | 0.4550 | -0.0886 | 0.1784 | 0.4822 | 0.0117 | 0.0819 |  |  |  |
| 95% confidence  interval | -0.1762  to  0.4895 | -0.0667  to  0.6393 | -0.04313 to  0.2267 | - 0.2228  to  0.4632 | 0.1158  to  0.6992 | -0.4350 to 0.2806 | -0.2050 to 0.3454 | 0.1294  to  0.7267 | -0.3801  to  0.4000 | -0.2970  to  0.4386 |  |  |  |
| *P value* | *0.309* | *0.096* | *0.498* | *0.456* | *0.0089* | *0.6415* | *0.3454* | *0.0081* | *0.9538* | *0.6670* |  |  |  |
| P value  summary | ns | ns | ns | ns | ** | ns | ns | ** | ns | ns |  |  |  |
| Δ: delta, difference between pre and post-transplantation values; MMP: matrix metalloproteinase; TIMP: MMP inhibitor; COL: collagen; PDGF: platelet-derived growth factor; r: Spearman's correlation coefficient; ns: non-significant | | | | | | | | | | | | | |
