# Supplementary material for: Autologous hematopoietic stem cell transplantation promotes connective tissue remodeling in systemic sclerosis patients
Source: Arthritis Res Ther. 2022 Apr 29;24:95. doi: 10.1186/s13075-022-02779-w (PMC9052524; doi:10.1186/s13075-022-02779-w)
Supplement: Supplementary file 2 — Additional file 2: Table S2: Correlations between serum molecules and modified Rodnan’s skin score (mRSS). [file 13075_2022_2779_MOESM2_ESM.docx]

| Correlation  (Spearman) | ΔmRSS  vs  ΔMMP-1 | ΔmRSS  vs  ΔMMP-3 | ΔmRSS  vs  ΔMMP-12 | ΔmRSS  vs  ΔMMP-13 | ΔmRSS  vs  ΔTIMP-1 | ΔmRSS  vs  ΔCOL1A1 | ΔmRSS  vs  ΔCOL4A1 | ΔmRSS  vs  ΔFGF-1 | ΔmRSS  vs  ΔPDGF-AA | ΔmRSS  vs  ΔPDGF-BB | ΔmRSS  vs  ΔS100A9 |  |  |  |
| --- | --- | --- | --- | --- | --- | --- | --- | --- | --- | --- | --- | --- | --- | --- |
| r | 0.6051 | -0.2619 | 0.1097 | -0.2353 | 0.1948 | -0.3529 | -0.1657 | -0.0446 | 0.4701 | 0.3115 | 0.4913 |  |  |  |
| 95 % confidence  interval | 0.3744 to 0.7653 | -0.5197 to 0.0395 | -0.1951 to 0.3951 | -0.4987 to 0.0677 | -0.1099 to 0.4661 | -0.5892 to -0.0609 | -0.4423 to 0.1395 | -0.3385 to 0.2572 | 0.1998 to 0.6740 | 0.0145 to 0.5580 | 0.2261 to 0.6888 |  |  |  |
| P value | 0.00001 | 0.079 | 0.468 | 0.115 | 0.194 | 0.016 | 0.271 | 0.768 | 0.001 | 0.035 | 0.001 |  |  |  |
| P value  summary | ***** | ns | ns | ns | ns | ** | ns | ns | *** | * | *** |  |  |  |
| Δ: Δ: delta, difference between pre and post-transplantation values; MMP: matrix metalloproteinase; TIMP: MMP inhibitor; COL: collagen; PDGF: platelet-derived growth factor; r: Spearman's correlation coefficient; ns: non-significant. | | | | | | | | | | | | | | |
