# Supplementary material for: Autologous hematopoietic stem cell transplantation promotes connective tissue remodeling in systemic sclerosis patients
Source: Arthritis Res Ther. 2022 Apr 29;24:95. doi: 10.1186/s13075-022-02779-w (PMC9052524; doi:10.1186/s13075-022-02779-w)
Supplement: Supplementary file 3 — Additional file 3: Table S3: Serum levels of molecules in SSc patients clustered according to severity of skin involvement. [file 13075_2022_2779_MOESM3_ESM.docx]

|  | **Baseline** | | |  | **After AHSCT** | | |  | **Delta** | | |
| --- | --- | --- | --- | --- | --- | --- | --- | --- | --- | --- | --- |
| **Marker** | **mRSS ≤ 20** | **mRSS > 20** | **P value** |  | **mRSS ≤ 20** | **mRSS > 20** | **P value** |  | **mRSS ≤ 20** | **mRSS > 20** | **P value** |
| MMP-1 | 1923 (838) | 3156 (1998) | *0.226* |  | 1242 (547) | 1820 (1147) | *0.134* |  | -680.6 (914) | -1336 (1034) | *0.1623* |
| MMP-3 | 13033 (11648) | 7606 (8708) | *0.065* |  | 17362 (13706) | 6401 (3059) | ***0.007*** |  | 4329 (17017) | -1204 (9480) | *0.089* |
| MMP-12 | 223 (85.8) | 177 (85.8) | *0.179* |  | 243 (99.5) | 159 (63) | ***0.014*** |  | 19.91 (138.9) | -18.06 (74.75) | *0.3656* |
| MMP-13 | 524 (259) | 371 (193) | ***0.039*** |  | 597 (274) | 346 (89) | ***0.002*** |  | 72.54 (361.7) | -24.67 (210.1) | *0.0989* |
| S100A9 | 1229 (2331) | 1585 (3006) | *0.151* |  | 512 (856) | 432 (415) | *0.680* |  | -716.2 (1505) | -1653 (3025) | *0.3943* |
| COL1A1 | 18615 (16313) | 18019 (11642) | *0.912* |  | 33254 (21988) | 46143 (29513) | *0.394* |  | 14640 (25009) | 28124 (27677) | *0.3420* |
| COL4A1 | 473 (198) | 711 (309) | ***0.034*** |  | 497 (151) | 587 (211) | *0.236* |  | 24.6 (100.3) | -123.7 (305.2) | *0.1344* |
| TIMP-1 | 131148 (81031) | 158403 (60314) | *0.325* |  | 115851 (64442) | 135344 (27043) | *0.288* |  | -15297 (48850) | -23059 (40429) | *0.6562* |
| PDGF-AA | 2625 (1627) | 3383 (1310) | *0.192* |  | 2202 (1289) | 2331 (777) | *0.748* |  | -423 (836.4) | -1053 (1048) | *0.0635* |
| PDGF-BB | 3427 (1937) | 5742 (4701) | ***0.038*** |  | 3325 (2043) | 3731 (1132) | *0.567* |  | -102 (859) | -2011 (4780) | ***0.0486*** |
| FGF-1 | 38.0 (21.1) | 35.1 (30.3) | *0.225* |  | 41.4 (20.5) | 24.7 (6.2) | ***0.005*** |  | 3.334 (27.98) | -10.41 (29.61) | *0.1214* |
| Mean (standard deviation) values of percentage of serum concentrations of connective tissue-related molecules in patients with baseline mRSS values ≤ 20 (n = 11) or > 20 (n = 16), measured at baseline and after AHSCT. Delta: differences between baseline and after AHSCT serum levels. P values within baseline and after AHSCT columns correspond to comparisons between higher or lower mRSS. Analyses used Student’s t-test or Mann-Whitney test, according to normality distribution status. SSc: systemic sclerosis; AHSCT: autologous hematopoietic stem cell transplantation; mRSS: modified Rodnan’s Skin Score; MMP: matrix metalloproteinase; TIMP: metalloprotease inhibitor; PDGF: platelet-derived growth factor; FGF: fibroblast growth factor; COL: collagen. | | | | | | | | | | | |
