# Supplementary material for: Autologous hematopoietic stem cell transplantation promotes connective tissue remodeling in systemic sclerosis patients
Source: Arthritis Res Ther. 2022 Apr 29;24:95. doi: 10.1186/s13075-022-02779-w (PMC9052524; doi:10.1186/s13075-022-02779-w)
Supplement: Supplementary file 4 — Additional file 4: Table S4: Immunohistochemistry (skin) results in SSc patients clustered according to severity of skin involvement. [file 13075_2022_2779_MOESM4_ESM.docx]

|  | **Baseline** | | |  | **After AHSCT** | | |  | **Delta** | | |
| --- | --- | --- | --- | --- | --- | --- | --- | --- | --- | --- | --- |
| **Marker** | **mRSS ≤ 20** | **mRSS > 20** | **P value** |  | **mRSS ≤ 20** | **mRSS ≤ 20** | **mRSS ≤ 20** |  | **mRSS ≤ 20** | **mRSS > 20** | **P value** |
| MMP-1 | 1.98 (1.56) | 3.01 (2.40) | *0.206* |  | -0.43(1.461) | -0.43(1.461) | -0.43(1.461) |  | -0.43(1.461) | -0.01156 (3.07) | *0.2361* |
| MMP-2 | 1.48 (1.11) | 1.76(0.89) | *0.279* |  | 2.245 (3.31) | 2.245 (3.31) | 2.245 (3.31) |  | 2.245 (3.31) | 1.147 (1.645) | *0.2523* |
| MMP-3 | 1.00 (0.61) | 1.24 (0.93) | *0.446* |  | 0.1724 (0.438) | 0.1724 (0.438) | 0.1724 (0.438) |  | 0.1724 (0.438) | 0.9787 (0.114) | ***0.027*** |
| MMP-9 | 1.41 (1.12) | 1.45 (1.03) | *0.911* |  | 0.2373 (1.026) | 0.2373 (1.026) | 0.2373 (1.026) |  | 0.2373 (1.026) | 1.068 (1.187) | *0.0712* |
| TIMP-1 | 2.82 (3.21) | 3.52 (3.18) | *0.208* |  | 4.828 (5.744) | 4.828 (5.744) | 4.828 (5.744) |  | 4.828 (5.744) | 0.5129 (4.902) | *0.0533* |
| NF-κB | 0.1850 (0.253) | 0.1093 (0.078) | *0.764* |  | -0.1367 (0.224) | -0.1367 (0.2240) | -0.1367 (0.2240) |  | -0.1367 (0.2240) | -0.071 (0.0719) | *0.7029* |
| α-SMA | 0.75 (0.65) | 0.74 (0.77) | *0.728* |  | 0.3879 (1.232) | 0.3879 (1.232) | 0.3879 (1.232) |  | 0.3879 (1.232) | -0.2432 (0.7646) | *0.1594* |
| TGF-β | 0.054 (0.075) | 0.015 (0.017) | ***0.050*** |  | 0.00363 (0.073) | 0.00363 (0.073) | 0.00363 (0.073) |  | 0.00363 (0.073) | 0.0045 (0.0265) | *0.4134* |
| Mean (standard deviation) values of percentage of area marked by immunohistochemistry in patients with baseline mRSS values ≤ 20 (n = 11) or > 20 (n = 16), measured at baseline and after AHSCT. Delta: differences between baseline and after AHSCT skin expressions. P values within baseline and after AHSCT columns correspond to comparisons between higher or lower mRSS. Data were analyzed by Student’s t-test or Mann-Whitney test, according to normality distribution status. SSc: systemic sclerosis; AHSCT: autologous hematopoietic stem cell transplantation; mRSS: modified Rodnan Skin Score; MMP: matrix metalloproteinase; TIMP: metalloprotease inhibitor; NF-κB: nuclear factor κB; α-SMA: alpha-smooth muscle actin; TGF-β: transforming growth factor. | | | | | | | | | | | |
