# Supplementary material for: Autologous hematopoietic stem cell transplantation promotes connective tissue remodeling in systemic sclerosis patients
Source: Arthritis Res Ther. 2022 Apr 29;24:95. doi: 10.1186/s13075-022-02779-w (PMC9052524; doi:10.1186/s13075-022-02779-w)
Supplement: Supplementary file 5 — Additional file 5: Table S5: Summary of transplanted-induced changes in connective tissue and fibrosis-related molecules in the serum and skin of systemic sclerosis patients. [file 13075_2022_2779_MOESM5_ESM.docx]

| **Marker** | **Serum** | **Skin** |
| --- | --- | --- |
| MMP-1 | Decreased | No changes |
| MMP-2 | - | Increased |
| MMP-3 | No changes | Increased |
| MMP-9 | - | Increased |
| MMP-12 | No changes | - |
| MMP-13 | No changes | - |
| TIMP-1 | Decreased | Increased |
| COL1 | Increased | Decreased * |
| COL4 | No changes | - |
| α-SMA | - | No changes |
| NF-κB | - | Decreased |
| TGF-β | - | No changes |
| FGF-1 | No changes | - |
| S100A9 | Decreased | - |
| PDGF-AA | Decreased | - |
| PDGF-BB | Decreased | - |
| *Density of total collagen in skin, evaluated by Picrosirius Red staining | | |
